# Long Non-Coding RNA in Vascular Disease and Aging

**DOI:** 10.3390/ncrna5010026

**Published:** 2019-03-19

**Authors:** Diewertje I. Bink, Noelia Lozano-Vidal, Reinier A. Boon

**Affiliations:** 1 Department of Physiology, Amsterdam Cardiovascular Sciences, Amsterdam UMC, VU University, 1081HV Amsterdam, The Netherlands; d.bink1@vumc.nl (D.I.B.); n.lozanovidal@vumc.nl (N.L.-V.); 2 Institute of Cardiovascular Regeneration, Goethe University, 60596 Frankfurt am Main, Germany; 3 German Center for Cardiovascular Research (DZHK), Partner Site Rhein-Main, 13347 Berlin, Germany

**Keywords:** aging, vascular disease, lncRNA

## Abstract

Cardiovascular diseases are the most prominent cause of death in Western society, especially in the elderly. With the increasing life expectancy, the number of patients with cardiovascular diseases will rise in the near future, leading to an increased healthcare burden. There is a need for new therapies to treat this growing number of patients. The discovery of long non-coding RNAs has led to a novel group of molecules that could be considered for their potential as therapeutic targets. This review presents an overview of long non-coding RNAs that are regulated in vascular disease and aging and which might therefore give insight into new pathways that could be targeted to diagnose, prevent, and/or treat vascular diseases.

## 1. Introduction

The sequencing of the human genome revealed that the majority of the human genome does not code for proteins [1,2]. The large number of non-protein coding transcripts were assumed to be junk RNA, but nowadays it is recognized that these non-coding RNAs (ncRNAs) have important cellular functions [3]. Non-coding RNAs can be divided into small non-coding RNAs, like microRNAs (miRNA), small nucleolar RNAs, piwi-interacting RNAs, and larger long non-coding RNAs (lncRNAs), which are transcripts larger than 200 nt [3,4]. These lncRNAs are a heterogeneous group of transcripts that can exert different functions in cells dependent on their cytoplasmic or nuclear localization in the cell. In the nucleus, they can act as transcription factors, chromatin modifiers, ribonucleoprotein (RNP) complexes or they can influence splicing. In the cytoplasm, they can influence RNA stability or bind to miRNAs, thereby regulating the mRNA targets of the miRNA [3].

### 1.1. Age and Cardiovascular Disease

Many lncRNAs are found to be regulated during aging and disease. Discovering the role of age-regulated lncRNAs will become increasingly important as life expectancy is increasing [5]. People aged >85 years have been the most rapidly expanding segment of the population over the past decades [5], and since this group is the most susceptible to disease, this will lead to increasing health costs. In the elderly, cardiovascular diseases are the most prominent cause of death [6]. As most cardiovascular diseases are age-dependent, cellular changes that appear with aging are likely to influence the susceptibility to developing cardiovascular diseases. With aging, the vessel walls stiffen, leading to a reduction of dampening of the pulsatile flow and influencing blood pressure and endothelial activation [7,8]. Development of atherosclerosis is already seen at a young age, but as the body ages, the plaques become bigger and less stable, increasing the risk of thrombus formation and infarction [9]. Although aging normally reduces the amount of angiogenesis [10,11], in diabetic patients there is an increase of angiogenesis in the course of the disease in tissues like the retina due to hyperglycemia-induced macro- and microvascular damage [12]. This increased angiogenesis is, however, unwanted since the capillarization occurs in a disordered fashion by highly-permeable vessels, leading to retinal edema, apoptosis of the surrounding cells, and subsequent hypoperfusion and tissue hypoxia.

### 1.2. LncRNAs in Vascular Disease

Long non-coding RNAs are able to influence different processes in the vascular system. They can influence vessel development, outgrowth, remodeling, tube formation, and angiogenesis by regulation of cellular processes like proliferation, apoptosis, adhesion, migration, and differentiation of endothelial cells (ECs) and vascular smooth muscle cells (SMCs) [13]. This will affect vascular diseases like atherosclerosis, aneurysms, hyper- or hypotension, retinopathies or diseases with vascular components like diabetes. Inflammation also plays an important role in atherosclerosis, so lncRNAs influencing the inflammatory system may also be important for vascular pathologies [14]. The role of lncRNAs in the different vascular cell types has been reviewed before by Simion et al. [13,15] and other reviews have summarized the role of lncRNAs in atherosclerosis [14,16], angiogenesis [15], aneurysms [17], and diabetes [18,19].

## 2. LncRNA Function in Vascular Diseases

In this review, we will discuss the role of ten lncRNAs which are implicated in vascular diseases and changed with aging and we arranged them based on their function. These lncRNAs also have functions in other cell types, but for now we will only consider the functions studied in the vasculature and in vitro studies in vascular ECs, perivascular/mural cells, such as fibroblasts, SMCs and pericytes, and cells of the immune system that can affect endothelial activation. Long non-coding RNAs can be divided into different groups based on their cellular location (e.g., cytoplasmic or nuclear), genomic location (e.g., sense, antisense, intronic, inter-genetic), mechanism of functioning (e.g., transcriptional regulation, post-transcriptional regulation or other functions) or mechanism of action. Here, we will divide the lncRNAs into four archetypes based on their mechanism of action [20,21]. One lncRNA can be part of more than one of these groups.

- Decoy lncRNA: These are RNP-binding lncRNAs, which inhibit the biological function of the RNP by preventing them from binding to their targets. This group also includes competing endogenous RNAs (ceRNAs) which bind to miRNAs and are often claimed to “sponge” off the miRNA. However, because lncRNAs are expressed at low levels and miRNAs are expressed at high levels, it is not completely clear how a lncRNA sponge would have such a big effect on miRNA levels [3].
- Guide lncRNA: These lncRNAs bind to a transcription factor or a chromatin modifier and guide it to the target promoter thereby altering the transcription of the target gene.
- Scaffold lncRNA: These lncRNAs bind to several RNPs, bringing them together to form ribonucleoprotein complexes, which in some cases can lead to transcriptional activation or repression.
- Enhancer lncRNAs: these lncRNAs are transcribed from enhancer regions and bring together enhancers and promoters in the genome through chromosomal looping to activate expression. We did not find published evidence for enhancer lncRNAs that play a role in vascular aging.

A fifth archetype has also been described, called signal lncRNAs. This group of lncRNAs are transcribed in response to a certain stimulus or stimuli in a cell-type-specific manner by RNA polymerase II, but since all lncRNAs are in the end stimulated in one way or the other and subsequently exert their action via one of the mechanism described beneath, we will not discuss these as a separate group.

### 2.1. Decoy lncRNAs

**Figure 1:**
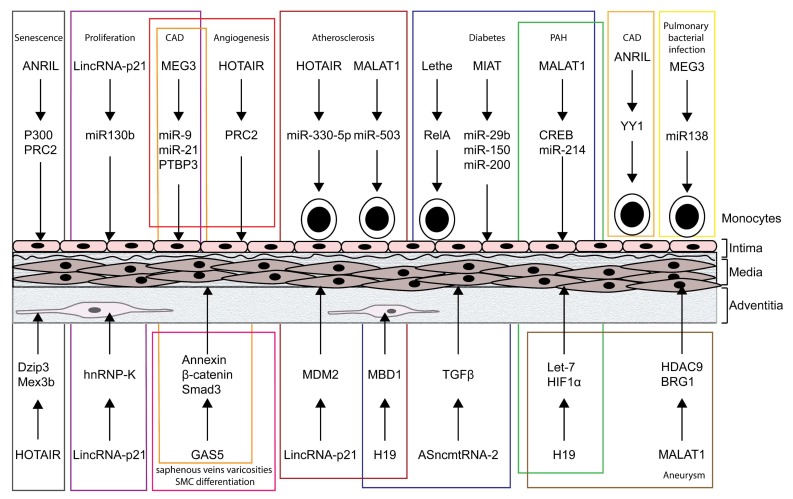
Long non-coding RNAs (LncRNAs) associated with vascular disease and age, and exerting a decoy, guide or scaffold function in endothelial cells, vascular smooth muscle cells, fibroblasts and/or immune cells. CAD: coronary artery disease; PAH: pulmonary arterial hypertension.

Maternally expressed gene 3 (*Meg3*) is a nuclear lncRNA implicated in different cardiovascular diseases and found to be upregulated in old human cardiac atria, mouse livers, and senescent human umbilical vein endothelial cells (HUVECs) compared to their young controls [22,23]. Lower *Meg3* expression is found in patients with coronary artery disease (CAD) [24], pulmonary arterial hypertension (PAH) [25], and metabolic syndrome [26], but increased expression was found in diabetes type 2 [27] and heart failure patients [28]. One of the molecular mechanisms by which *Meg3* can alter cellular functions is by its decoy function. Maternally expressed gene 3 is thought to suppress miR-21 expression in ECs, which is found to be upregulated in CAD patients, and thereby influence RhoB, PTEN, and collagen expression and EC proliferation [24] (Figure 1). The effect of *Meg3* on EC proliferation and capillary formation has, however, also been partially attributed to the binding to miR-9 [29]. Hypoxia, an important trigger for angiogenesis, increases the expression of *Meg3* [30]. In cardiomyocytes, it has been found that this leads its binding to the protein FUS and increases apoptosis [28], but it has not yet been published if the same mechanism applies to ECs or SMCs. Inflammation has also been found to be influenced by *Meg3*. The splice variant *Meg3–4* is found to bind to the microRNA miR-138, thereby increasing the miR-138 target pro-inflammatory cytokine interleukin 1 beta in macrophages [31].

Another nuclear lncRNA induced by hypoxia is metastasis associated lung adenocarcinoma transcript 1 (*MALAT1*), also known as *NEAT2* [30,32]. The expression of MALAT1 is reduced in replicative senescence [33] and in atherosclerotic plaques [34], while its increased expression is found in diabetes type 2 patients [27]. In addition, genetic variations in the *MALAT1* gene are associated with decreased risk for coronary atherosclerotic disease [35] and PAH [36]. Not only in humans, but also in diabetic mice and rats and stressed retinal cells, *MALAT1* expression is increased [37,38]. The lncRNA *MALAT1* was found to bind to CREB, thereby inhibiting its dephosphorylation by PP2A and the resulting continuous CREB signaling leads to abnormal cell viability and hyperproliferation. In atherosclerotic mice, *MALAT1* seems to target miR-503, leading to an increase in FGF2 in monocytes [34]. In HUVECs, the *MALAT1* variant that showed a reduced PAH risk was found to interact with miR-214, thereby increasing the expression of X box-binding protein 1, and consequently *MALAT1* could inhibit EC proliferation and migration [36].

The lncRNA myocardial infarction associated transcript (*MIAT*), also called *Gomafu*, is downregulated in senescent fibroblasts compared to early passage proliferation fibroblasts [39]. Like *MALAT1*, *MIAT* is also upregulated in the retinas and peripheral blood mononuclear cells of diabetes patients [27,40], and is furthermore upregulated in peripheral blood leukocytes of ischemic stroke patients [41]. In addition, *MIAT* is found to be increased in a mouse diabetes model and in vitro after high glucose treatment [40,42], which is at least partially the effect of increased binding of the protein complex NF-κB to the *MIAT* promotor. Overexpression of *MIAT* suppresses miR-29b which leads to decreased cell viability and increased apoptosis. Knockdown of *MIAT* reverses the high glucose-induced apoptosis and decreased cell activity [42]. Furthermore, *MIAT* is also a decoy for miR-150 and miR-200a, thereby reducing the repression of VEGF by these miRNAs [40,43]. The increased *MIAT* expression in diabetic patients could therefore be (partially) responsible for the increase in VEGF and pathological angiogenesis.

The lncRNA *H19* is a cytoplasmic lncRNA [44,45] which is highly expressed in the embryonic stage, but its expression decreases shortly after birth [46,47,48]. However, in some vascular diseases, the expression of *H19* increases again. Increased expression has been shown in aortic aneurysms [49], atherosclerotic lesions [48], in calcific aortic valves [50], and in plasma levels of CAD and ischemic stroke patients [51,52]. On the contrary, decreased levels in ECs of carotid atherosclerotic plaques have been found by Hofmann et al. [45]. Genetic variation in *H19* has also been described to lead to an increased risk for CAD [53] and ischemic stroke [54]. In the cardiovascular system, the binding of *H19* to the let-7 family seems to be important [55,56]. In PAH, the *H19*/let-7 interaction was found to lead to an increase in angiotensin receptor AT1R levels and increase in proliferation. A knockdown of H19 in mice could reduce the pulmonary artery remodeling in the PAH model [56]. Moreover, *H19* was also suggested to bind to HIF1α, thereby retaining HIF1α in the cytoplasm, and in addition, H19 facilitated the binding of transcription factor Sp1 to the HIF1α promotor, increasing HIF1α expression [49]. Subsequently, HIF1α binds to mouse double minute 2 homolog (MDM2), thereby reducing MDM2 binding to tumor protein p53which would normally lead to p53 degradation, thereby leading to a decrease in SMC proliferation and an increase in apoptosis and aneurysm progression.

Another lncRNA that is inhibited after transition from embryonic to adult stage is *lincRNA-p21* [57]. Also genetic variation in *lincRNA-p21* has shown to influence CAD and myocardial infarction [58], and levels are decreased in coronary tissue from CAD patients [59] and in atherosclerotic plaques [60], but increased in thoracic aorta aneurysm [61] and diabetes type 2 patients [27]. The nuclear lncRNA lincRNA-p21 is regulated by p53 [62]. In SMCs and macrophages *lincRNA-p21* interacts with MDM2, which would otherwise bind to p53 and facilitate p53 degradation [59]. Silencing of *lincRNA-p21* in SMCs or mouse carotid increases the binding of MDM2 to p53, but decreases the binding between p300 and p53, leading to reduced transcription of p53 target genes, such as *PUMA* and *Bax*, and thereby reducing apoptosis and increasing intima-media thickness. In mouse ECs, *lincRNA-p21* is thought to act as a ceRNA for miR-130b, thereby inhibiting proliferation [63].

The lncRNA HOX Antisense Intergenic RNA (*HOTAIR*) is an antisense lncRNA flanked by *HOXC11* and *HOXC12*. It is expressed both in the cytoplasm and the nucleus and found to be upregulated in senescent fibroblasts [64,65]. Silencing of *HOTAIR* reduced the levels of senescent markers and lowered β-galactosidase activity. The expression of *HOTAIR* is decreased in sporadic thoracic aortic aneurysm tissue [66], ECs for atherosclerotic plaques [67], and in the peripheral blood lymphocytes of atherosclerosis patients [68], but increased in patients with diabetes [27] and congenital heart disease [69]. In atheromatous plaques hypermethylation of CpG sites in the *HOTAIR* gene was found [70]. As inflammation is an important aspect of atherosclerosis, the function of *HOTAIR* has been studied in monocyte/macrophage cell lines. In a human monocytic cell line *HOTAIR* expression was found to be increased upon ox-LDL treatment and silencing of *HOTAIR* reduced cholesterol levels, reactive oxygen species (ROS) production and pro-inflammatory cytokines through the interaction with miR-330-5p [71]. However, ox-LDL has shown to decrease *HOTAIR* expression in a mouse macrophage cell line and overexpression of *HOTAIR* led to increases in anti-inflammatory and adipose gene expression and a decrease in pro-inflammatory and lipogenesis gene expression [68]. In this cell line *HOTAIR* increased the expression of the RNA-binding protein FXR1 and thereby reduced inflammatory response by inhibition of the NF-κB pathway. It is not investigated if this process involved a decoy function of *HOTAIR*. However, since both the human and mouse cell line are immortalized, they might have an altered cellular phenotype that could influence the acquired results and might not resemble the in vivo situation.

Compared to most lncRNAs mentioned above, there is relatively little known about the lncRNA *Lethe*. Long non-coding RNA *Lethe* is a pseudogene of *Rps15a* and is found to be downregulated with age in mice [72]. It is found to be decreased in the wounds of diabetic mice [73] and is regulated by glucose pro-inflammatory cytokines via NF-κB. Lethe binds to the NF-κB subunit RelA and thereby represses NF-κB binding to chromatin, creating a negative feedback loop and reducing ROS production [72,73].

Growth arrest specific 5 (*GAS5*) was found to be upregulated with age in the mouse hippocampus [74], but downregulated with age in the rest of the brain and in mouse ovaries [75]. It is unknown what the effect of aging is on *GAS5* levels in the cardiovascular system. Polymorphisms of *GAS5* have been associated with atherosclerosis risk [76] and the expression is increased in rat and patient atherosclerotic plaques [77]. In patients with hypertension, type 2 diabetes, CAD, and primary varicose great saphenous veins, the expression was, however, decreased [27,78,79,80]. Knockdown of *GAS5* in hypertensive rats aggravates the hypertension phenotype, including further increases in blood pressure, vascular remodeling, formation of retinal angiogenesis, and vascular leakage [78]. These effects are thought to be the result of an interaction between *GAS5* and β-catenin, which decreases β-catenin nuclear translocation, and thereby the expression of its target genes. Correspondingly, in rats with CAD an abnormally activated Wnt/β-catenin signaling pathway was found and could be decreased by *GAS5* overexpression [80]. In vein SMCs, *GAS5* was found to bind to Annexin A2, influencing proliferation and thereby possibly the pathogenesis of great saphenous veins varicosities [79]. In addition, *GAS5* has also been shown to bind to Smad3 in SMCs, which reduces the binding of Smad3 to gene promoters normally activated by TGF-beta signaling, which results in the inhibition of TGF-beta/Smad3-mediated differentiation of SMCs [81].

### 2.2. Guide lncRNAs

Besides acting as a decoy lncRNA, *Meg3* can also function as a guide lncRNA. The lncRNA *Meg3* binds to both PTBP1 and PTBP3 [82,83]. The RNA-binding protein PTBP3 has been shown to bind to the promoters of p53 targets in HUVECs and knockdown influences the transcription of these targets. The EC function, like proliferation and apoptosis, is compromised by knockdown of either *Meg3* or PTBP3, an effect which is not seen after PTBP1 knockdown, suggesting an important role of the *Meg3*–PTBP3 binding in ECs. An effect of *Meg3* on p53 target signaling has also been shown in pulmonary SMCs [25] and hepatoma cells [84]. Besides *Meg3*, *lincRNA-p21* also affects p53 target expression. The binding of *lincRNA-p21* to hnRNP-K in MEFs leads to promotor binding to and repression of cell death-repressing genes which are part of the p53 transcriptional response [62].

The lncRNA *MALAT1* also has a guide function in addition to its decoy function. It forms a complex with HDAC9 and BRG1, which binds to PRC2 and thereby increases H3K27me3 marks on contractile protein gene promotors and represses their expression [85]. In SMCs of aortas from an aneurysm mouse model, increased expression and colocalization of *HDAC*9, *BRG1,* and *MALAT1* was found and deletion of *MALAT1* or *HDAC9* restored the contractile protein expression and inhibited aneurysm growth. In addition, *HOTAIR* also interacts with PRC2, thereby inducing H3K27-methylation which results in suppression in gene expression, for example of *HOXD* [86,87,88]. Since *HOXD* genes are found to affect angiogenesis [89,90], this transcriptional regulation might be important for the effect of *HOTAIR* on vascular diseases. On the other hand, *H19* induces H3K9me3 modifications by interacting with MBD1 [91]. This leads to transcriptional control over several genes, including *IGF2* which is implicated in diabetes and atherosclerosis [92,93].

A lncRNA that has no decoy function, but functions as a guide lncRNA is antisense non-coding RNA in the INK4 locus (*ANRIL*). The expression of *ANRIL* increases with age in rodents [87], but decreases in expression in later passages of some cell lines [94,95]. Genetic susceptibility of the *ANRIL* locus has been associated with CAD [96,97], but also with atherosclerosis [98], intracranial aneurysms [99], and type 2 diabetes [100]. In fibroblasts, *ANRIL* has shown to bind to the PRC2 complex, thereby influencing binding of PRC2 to the p15^INK4B^ locus [101]. Loss of *ANRIL* reduces PRC2 binding to the p15^INK4B^ locus and increases its expression, thereby inducing cellular senescence. The binding to PRC2 and in addition p300 is also thought to increase VEGF expression, which subsequently influences tube formation of retinal ECs in vitro and retinal microvascular permeability in vivo [102]. Furthermore, *ANRIL* seems to bind to the transcription factor YY1 after TNFα induction, thereby inducing IL6 and IL8 transcription [103]. Since a correlation between *ANRIL* levels and IL6 and IL8 levels was found in CAD patients and elevated IL8 levels are associated with CAD risk [104], *ANRIL* may be a potential therapeutic target for CAD.

### 2.3. Scaffold lncRNAs

Scaffold lncRNAs bind to several RNPs, bringing them together to form ribonucleoprotein complexes. The lncRNA *HOTAIR* brings together PRC2 with the LSD1/CoREST/REST complex increasing H3K27me3 and decreasing H3K4me2 [87]. Two of the target genes shown to be affected by *HOTAIR* knockdown, *SIRT2* and *LRP1B*, have been implicated in atherosclerosis [105,106]. The scaffolding of LSD1 might also affect aneurysm formation, as LSD1 has also been reported to affect collagen I expression in osteoblasts [107] and silencing of *HOTAIR* in HASMCs has been shown to suppress collagen I expression and leads to weakening off the aortic wall [66]. In addition, *HOTAIR* also acts as a scaffold between E3 ubiquitin ligases Dzip3 and Mex3b and their corresponding substrates Ataxin-1 and Snurportin which might affect senescence [64].

The binding of *ANRIL* to PRC1 and PRC2 has not only been described as two separate guiding mechanisms, but scaffold action binding of both complexes has also been proposed [20,95,101].

### 2.4. Other lncRNAs Regulated in Vascular Diseases and Aging

A lncRNA which is induced by aging in mouse aorta and old passage HUVECs [108], but with an unknown functional mechanism is the antisense noncoding mitochondrial RNA *ASncmtRNA-2*. This lncRNA is transcribed from mitochondrial DNA [109] and is upregulated in diabetic mouse kidneys and in vitro after high-glucose-induced ROS production [110]. Knockdown of *ASncmtRNA-2* decreases the expression of the pro-fibrotic factors TGFβ1 and fibronectin, concluding that *ASncmtRNA-2* might be important in diabetic glomerular fibrosis. Since TGFβ1 and fibronectin are known to be regulated in aneurysms and *ASncmtRNA-2* is expressed in aortas [111], it might be interesting to test if *ASncmtRNA-2* plays a role in aneurysm formation.

## 3. LncRNAs as Biomarkers

Although differential plasma levels of several lncRNAs have been found in patients with vascular diseases, it is still unclear if these lncRNAs are suitable as specific biomarkers, since they are often also implicated in other diseases. For example, increased *H19* levels in plasma are found in patients with CAD and it was an independent predictor even after adjustment for known cardiovascular risk factors [51]. However, *H19* was also suggested as a biomarker for breast cancer [112] and gastric cancer [113] after discovering increased H19 levels in plasma in these patients. Likewise, *GAS5* levels in plasma are found to be decreased in diabetes type 2 patients and are even further decreased in CAD patients [114], but *GAS5* levels in plasma are also decreased in non-small cell lung cancer patients [115].

Upregulated levels of *lincRNA-p21* in serum could be used to distinguish thoracic aortic aneurysm patients from healthy people [61]. Reduced levels are on the other hand found in CAD patients [54], so *lincRNA-p21* has been suggested as a potential biomarker atherosclerosis. However, decreased levels are also found in hepatitis B positive liver fibrosis patients [116] and in colorectal cancer patients [117].

The lncRNA *ANRIL* could act as a prognostic factor for a more specific group of patients, since higher levels in plasma are associated with in-stent restenosis compared to patients with stent without stenosis [113]. Care should, however, be taken in patients with a higher risk for breast cancer, since increased levels of *ANRIL* in plasma are also found in patients with breast cancer [118]. Single-nucleotide polymorphisms in *ANRIL* can also be used as a biomarker, since rs10757274 and rs2383206 are associated with an increased risk of CAD [119,120,121].

## 4. Conclusions

In summary, we have discussed several lncRNAs which are differentially regulated with aging and are implicated in vascular disease. As aging is an important risk factor for cardiovascular diseases, the lncRNAs implicated in both might be the most promising therapeutic targets. The mechanisms by which these lncRNA influence vascular pathologies are starting to be unraveled, but further research still needs to be done. With a deeper understanding of the mechanisms by which lncRNAs act, novel and effective targets might be identified for the development of therapeutic strategies for vascular diseases. This process may be hampered by the lack of sequence conservation in other species and be delayed because lncRNAs often have multiple transcript variants, which do not always have the same function. An additional limitation might be that some annotated lncRNAs are later on found to encode a short peptide which was overlooked in the past.

## Abbreviations

ANRIL	antisense non-coding RNA in the INK4 locus
CAD	coronary artery disease
ceRNAs	competing endogenous RNAs
ECs	endothelial cells
GAS5	Growth Arrest Specific 5
HOTAIR	HOX Antisense Intergenic RNA
HUVECs	human umbilical vein endothelial cells
lncRNA	long non-coding RNA
MALAT1	Metastasis Associated Lung Adenocarcinoma Transcript 1
MDM2	Mouse double minute 2 homolog
Meg3	Maternally expressed gene 3
MIAT	Myocardial infarction associated transcript
miRNA	microRNA
ncRNA	non-coding RNA
PAH	pulmonary arterial hypertension
RNP	ribonucleoprotein
ROS	reactive oxygen species
SMCs	smooth muscle cells.
